# Knowledge of Iron Deficiency Anaemia and Associated Attributes Among School Adolescents in Eastern India: A Cross-Sectional Evaluation

**DOI:** 10.7759/cureus.86955

**Published:** 2025-06-29

**Authors:** Bijit Biswas, Anuradha Gautam, G. Jahnavi, Richa Richa, Pratima Gupta, Saurabh Varshney

**Affiliations:** 1 Community and Family Medicine, All India Institute of Medical Sciences, Deoghar, IND; 2 Microbiology, All India Institute of Medical Sciences, Deoghar, IND; 3 Otolaryngology, All India Institute of Medical Sciences, Deoghar, IND

**Keywords:** adolescent, anaemia, iron deficiency, knowledge, school children, weekly iron and folic acid supplementation

## Abstract

Background: This study assessed knowledge related to iron deficiency anaemia (IDA), weekly iron and folic acid supplementation (WIFS), and school-based deworming among school-going adolescents in Eastern India, along with key associated attributes.

Methods: A cross-sectional survey was conducted among 843 students (Standards 8-10) from four government schools in Deoghar, Jharkhand, using multistage probability sampling. A validated Hindi questionnaire was used to assess knowledge alongside symptoms, clinical pallor, and anthropometry.

Results: Median knowledge scores were 14 (interquartile range (IQR): 7-18) for IDA and 9 (IQR: 6-11) for WIFS and deworming. IDA knowledge positively correlated with the number of anaemia-related symptoms (ρ = 0.269, p < 0.001); WIFS and deworming knowledge showed a similar trend (ρ = 0.276, p < 0.001). Participants reporting ≥ 3 symptoms had significantly higher IDA scores than those with none (mean rank difference = 217.5 vs. 69.8; p < 0.001), and WIFS scores (190.2 vs. 61.7; p < 0.001). Knowledge was significantly higher in older students (≥ 16 years vs. < 14 years: p < 0.001), higher grades (Standards 9 and 10 vs. Standard 8: p < 0.001), and those with literate parents (both p < 0.001). Boys had higher WIFS knowledge than girls (p < 0.001).

Conclusion: Higher symptom burden and demographic factors were associated with greater knowledge, underscoring the need for focused anaemia education among less informed subgroups in school settings.

## Introduction

Iron deficiency anaemia (IDA) continues to pose a significant public health challenge globally, particularly among adolescents in low- and middle-income countries. It is estimated that over 2 billion individuals are anaemic, with iron deficiency accounting for nearly half of all cases [1,2]. Adolescents are especially vulnerable due to rapid physical growth, poor dietary diversity, and in girls additional losses from menstruation [1,3]. The consequences of IDA include impaired cognitive and physical development, increased susceptibility to infections, and reduced academic and work productivity [1,3,4].

India bears a substantial share of this burden. According to the National Family Health Survey (NFHS-5), anaemia prevalence among adolescents aged 15-19 years rose from 55.8% to 59.1% in girls and from 30.2% to 31.1% in boys compared to NFHS-4 [5]. In Jharkhand, the prevalence is even higher: 65.8% in girls and 39.7% in boys [6]. To address this issue, the Government of India introduced the weekly iron and folic acid supplementation (WIFS) programme in 2012 and the National Deworming Day (NDD) initiative in 2015, targeting adolescents through schools and anganwadis [7,8]. These programs provide weekly iron-folic acid tablets, biannual albendazole, and health education. However, despite nationwide implementation, studies have reported poor awareness, low compliance, and persistent misconceptions about supplementation, especially in underserved areas [9-11].

While most existing studies have focused on adolescent girls or evaluated program effectiveness, few have explored knowledge levels and their correlates among adolescents in school settings [10-14]. This study was conducted to assess knowledge regarding IDA, WIFS, and deworming among school-going adolescents in Deoghar district of Jharkhand, and to examine associations with socio-demographic, clinical, and nutritional attributes. The findings aim to inform more targeted and context-specific adolescent anaemia prevention strategies.

## Materials and methods

Study design and setting

An analytical cross-sectional study was conducted among school-going adolescents enrolled in Standards 8-10 across four coeducational government schools in Deoghar district, Jharkhand, India. The study aimed to assess knowledge related to IDA and associated factors. To capture contextual variability, two administrative blocks, Devipur and Deoghar, were purposively selected from the 10 blocks comprising the district. According to school cluster mapping, Devipur included nine school clusters with an average of 24 schools per cluster (range: 19-26), while Deoghar had 17 clusters with an average of 22 schools (range: 9-45) [15,16]. From each block, two clusters were randomly selected, and one coeducational school was randomly chosen from each cluster.

Sample size and sampling technique

The sample size was calculated based on the assumption that 15.1% of participants would demonstrate correct knowledge about IDA, as previously reported by Subba et al. in a school-based study from Anantapur district, Andhra Pradesh [12]. With a 95% confidence level, 20% relative precision, and a design effect of 1.5 to account for cluster sampling, the minimum required sample size was calculated to be 817 using the Statulator online tool [17]. A total of 843 students were ultimately enrolled in the study.

Sample allocation to each school was performed using the probability proportionate-to-size (PPS) method. Within each selected school, students were identified using computer-generated random number lists produced via OpenEpi [18]. 12 lists, one for each class section, contained roll numbers ranging from 1 to 150. On the day of the survey, students were approached based on these lists until the target number was reached for each school.

Data collection tools and procedures

Following written informed consent, data were collected using a structured, self-administered questionnaire in Hindi. The tool was developed through an extensive literature review focusing on adolescent anaemia, the WIFS programme, and school-based deworming initiatives [2,7,10-12,14,19-21]. Face, content, and criterion validity were established through expert consultation.

The questionnaire consisted of three sections. The first section captured socio-demographic information, including age, sex, caste, religion, parental education, and per capita monthly income (PCMI). The second section assessed knowledge of IDA using 34 items across seven domains: symptoms, clinical signs, consequences, causes, prevention, dietary sources, and absorption-related factors. Each correct response was scored as ‘1’, and incorrect as ‘0’, yielding a total possible score of 0-34. This section demonstrated high internal consistency (Cronbach’s α = 0.894).

The third section included 20 items related to WIFS and deworming, covering recommended intake schedules, benefits, and common side effects of iron and albendazole tablets. Scoring followed the same binary format (range: 0-20), with satisfactory internal consistency (Cronbach’s α = 0.783) (Appendix 1).

On the day of data collection, students were selected using class-wise, computer-generated random number lists corresponding to school roll numbers. Participants also self-reported common anaemia-related symptoms such as fatigue, dizziness, loss of appetite, and difficulty concentrating. Clinical pallor was assessed by the study investigators under natural daylight through inspection of the lower palpebral conjunctiva.

Anthropometric measurements were performed following World Health Organization (WHO) protocols. Body weight was measured to the nearest 0.1 kg using calibrated analogue weighing scales, and height was recorded to the nearest 0.1 cm using a fixed, non-stretchable measuring tape. BMI was calculated and categorized using WHO BMI-for-age percentile charts into five groups: severe thinness (< 3rd percentile), thinness (3rd- < 15th), normal (15th- < 85th), overweight (85th- < 97th), and obesity (≥ 97th percentile) [22].

In addition to knowledge, data on attitudes toward anaemia and practices related to WIFS and deworming-such as perceived vulnerability, severity, and self-reported consumption of tablets-were also collected as part of the broader mixed-methods study. These findings are reported separately in a dedicated manuscript focused on programmatic barriers and enablers of iron and folic acid supplementation and deworming in school settings [9].

Statistical analysis

Data were entered in Microsoft Excel and analyzed using Jamovi software (version 2.3.26). Descriptive statistics were used to summarize participant characteristics and knowledge responses. The distribution of knowledge scores related to IDA, WIFS, and deworming was assessed using Q-Q plots and the Kolmogorov-Smirnov (K-S) test. As the scores were not normally distributed (K-S test, p < 0.001), non-parametric tests were employed for group comparisons. The Mann-Whitney U test was used for binary variables, and the Kruskal-Wallis test was applied for variables with more than two categories. Where applicable, Dunn’s post hoc test with Bonferroni correction was performed for multiple comparisons. Spearman’s rank correlation coefficient (ρ) was used to examine the relationship between knowledge scores and the number of anaemia-related symptoms. A two-tailed p-value < 0.05 was considered statistically significant.

## Results

Knowledge regarding IDA among participants varied widely, with correct responses ranging from 87 (10.3%) to 556 (66.0%). The least known item was the role of walking barefoot in increasing the risk of hookworm infestation (87; 10.3%), while the most commonly identified was that consuming iron-rich foods helps prevent anaemia (556; 66.0%). The median IDA knowledge score was 14 (interquartile range (IQR): 7-18; range: 0-33). For WIFS and deworming, correct responses ranged from 99 (11.7%) to 794 (94.2%). The least known item was that iron tablets can cause constipation (99; 11.7%), while the highest correct response was recorded for identifying the false statement that deworming tablets have no benefit (794; 94.2%). The median WIFS knowledge score was 9 (IQR: 6-11; range: 3-20) (Table 1).

**Table 1:** Distribution of the study participants as per their knowledge regarding IDA, WIFS, and deworming (n = 843) IFA: Iron folic acid; IDA: Iron deficiency anaemia; WIFS: Weekly iron and folic acid supplementation

Item	Variable	Correct Response	N (%)
Knowledge regarding IDA			
	What do you know about the symptoms of anaemia?		
K1	Feeling tired is a symptom of anaemia	True	472 (56.0)
K2	Anaemia can cause difficulty in concentrating	True	251 (29.8)
K3	Loss of appetite can be a sign of anaemia	True	324 (38.4)
K4	Passing worms in the stool can be related to anaemia	True	167 (19.8)
K5	People with anaemia may feel their heart beating fast (palpitations)	True	172 (20.4)
	How can you recognize someone who has anaemia?		
K6	People with anaemia often feel weak	True	519 (61.6)
K7	Anaemia can cause paleness in the skin, tongue, or palms	True	281 (33.3)
K8	Spoon-shaped nails (koilonychia) can be a sign of anaemia	True	177 (21.0)
K9	Anaemic people may fall sick more often	True	195 (23.1)
	What do you know about the effects of anaemia on adolescents?		
K10	Anaemia can affect your physical growth	True	334 (39.6)
K11	It can lead to poor performance in school	True	386 (45.8)
K12	Anaemia can reduce your ability to fight infections	True	276 (32.7)
K13	It may cause menstrual problems in girls	True	174 (20.6)
	What are the causes of IDA?		
K14	Not eating enough iron-rich foods can cause anaemia	True	448 (53.1)
K15	Infections like malaria or worm infestation can lead to anaemia	True	294 (34.9)
K16	Heavy bleeding during periods may cause anaemia	True	225 (26.7)
K17	Walking barefoot can increase the chance of getting worm infestation	True	87 (10.3)
K18	Not taking iron tablets (IFA) can lead to anaemia	True	166 (19.7)
K19	Not taking deworming medicine (Albendazole) can increase anaemia risk	True	264 (31.3)
	How can anaemia be prevented?		
K20	Eating iron-rich foods helps prevent anaemia	True	556 (66.0)
K21	Eating fruits rich in vitamin C helps the body absorb iron	True	418 (49.6)
K22	Taking iron tablets regularly can prevent anaemia	True	272 (32.3)
K23	Treating infections early helps in preventing anaemia	True	151 (17.9)
	What are some good sources of iron?		
K24	Green leafy vegetables (like spinach) are rich in iron	True	470 (55.8)
K25	Jaggery (gur) is a good source of iron	True	488 (57.9)
K26	Whole grains (like wheat, bajra) contain iron	True	462 (54.8)
K27	Fruits (like pomegranate, apple) are sources of iron	True	514 (61.0)
K28	Liver (animal source) contains iron	True	312 (37.0)
K29	Fish is a good source of iron	True	354 (42.0)
K30	Pulses and legumes (like lentils, rajma) are rich in iron	True	226 (26.8)
K31	Nuts (like almonds, groundnuts) contain iron	True	327 (38.8)
	What helps or harms iron absorption and adolescent needs?		
K32	Drinking tea or coffee after meals reduces iron absorption	True	535 (63.5)
K33	Eating vitamin C-rich foods (like lemon or amla) helps absorb iron	True	461 (54.7)
K34	Teenagers need more iron and folic acid than children or adults	True	415 (49.2)
Knowledge regarding WIFS and deworming			
	How should iron tablets (IFA) AND deworming tablets (albendazole) be taken in school?		
K35	How often should the iron tablet (IFA) given in school be taken?	Weekly	306 (36.3)
K36	When should the iron tablet (IFA) be taken?	After Food	318 (37.7)
K37	How often should the deworming tablet (albendazole) be taken in school?	Twice a year (Bi-annually)	474 (56.2)
	What are the benefits of taking iron tablets (IFA)?		
K38	Iron tablets (IFA) have no benefit	False	755 (89.6)
K39	Iron tablets help improve concentration	True	339 (40.2)
K40	Iron tablets help you feel healthier	True	496 (58.8)
K41	Iron tablets help in gaining weight	True	187 (22.2)
K42	Iron tablets reduce tiredness	True	261 (31.0)
	What are the benefits of taking deworming tablets (albendazole)?		
K43	Deworming tablets have no benefit	False	794 (94.2)
K44	Deworming tablets kill stomach worms	True	632 (75.0)
K45	Deworming tablets kill filaria parasites	True	353 (41.9)
	What are the common side effects of iron tablets (IFA)?		
K46	Iron tablets have no side effects	False	667 (79.1)
K47	Iron tablets can make stool look black	True	210 (24.9)
K48	Iron tablets can cause a metallic taste in mouth	True	267 (31.7)
K49	Iron tablets can make the stomach feel full	True	194 (23.0)
K50	Iron tablets can cause constipation	True	99 (11.7)
K51	Iron tablets can cause stomach pain	True	144 (17.1)
K52	Iron tablets can cause loose motion (diarrhoea)	True	123 (14.6)
K53	Iron tablets can cause nausea	True	162 (19.2)
K54	Iron tablets can cause headache	True	191 (22.7)

**Figure 1:**
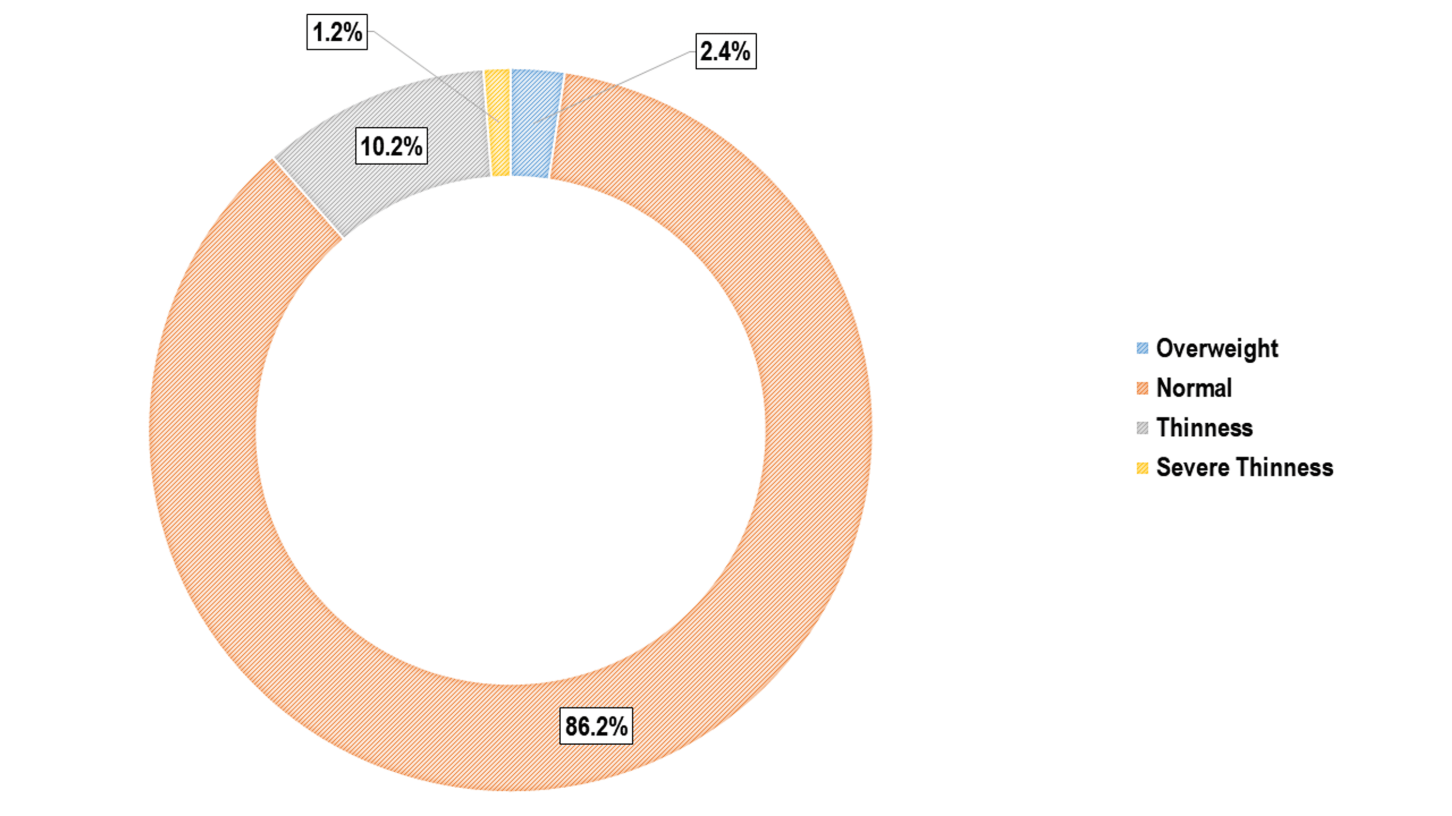
Doughnut diagram showing distribution of the study participants as per their nutritional status (n = 843)

**Figure 2:**
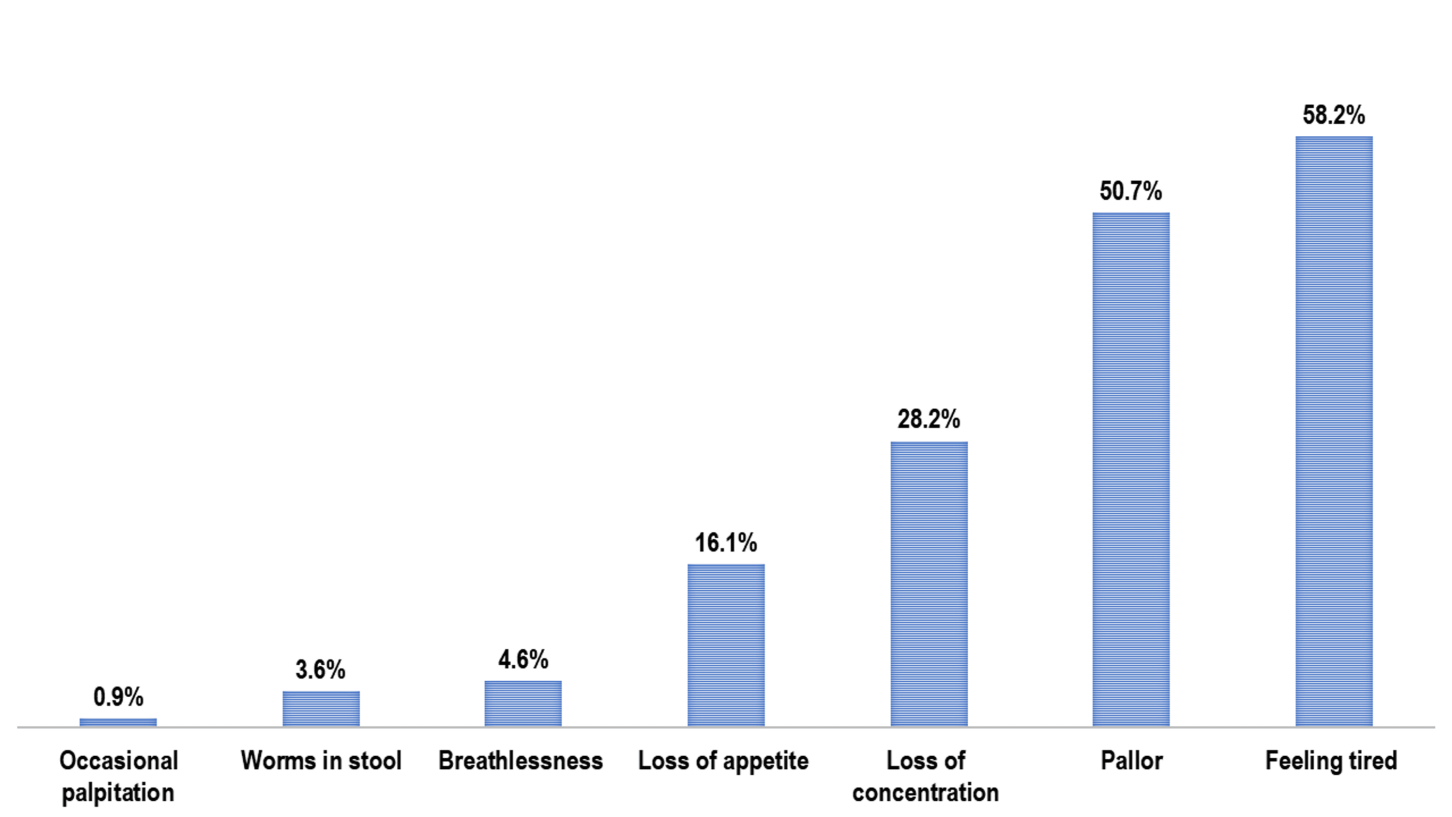
Bar chart showing distribution of the study participants as number of reported signs or symptoms of IDA (n = 843) IDA: Iron deficiency anaemia

The median age of participants was 15 years (IQR: 14-15; range: 12-18), with a slightly higher proportion of females (442 (52.4%)) than males (401 (47.6%)). The median PCMI was USD 15.3 (IQR: 10.2-21.4). Nutritional assessment showed that 116 (13.8%) participants were malnourished, including 86 (10.2%) with thinness, 10 (1.2%) with severe thinness, and 20 (2.4%) who were overweight (Figure 1). Fatigue was the most frequently reported symptom of anaemia (491; 58.2%), followed by difficulty concentrating (238; 28.2%) and loss of appetite (136; 16.1%). Clinical pallor was observed in 427 (50.7%) participants (Figure 2).

Knowledge regarding IDA was significantly associated with age, educational grade, caste, number of anaemia-related symptoms, parental literacy, diet type, and nutritional status (p < 0.001). Post hoc comparisons revealed significantly higher knowledge among participants aged 14 years (mean rank difference = 61.334; p = 0.029), 15 years (88.442; p = 0.001), and ≥ 16 years (135.550; p < 0.001) compared to those aged < 14 years. Those aged ≥ 16 years also outperformed 14-year-olds (74.216; p = 0.002) and 15-year-olds (47.108; p = 0.043). Students in Standards 9 and 10 had higher scores than those in Standard 8 (85.416; p < 0.001 and 55.536; p = 0.007, respectively). Participants from Other Backward Class (OBC) (133.138; p < 0.001), Scheduled Caste (SC) (100.716; p < 0.001), and Scheduled Tribe (ST) groups (133.283; p = 0.018) scored significantly higher than the General category. IDA knowledge increased with the number of anaemia-related symptoms: Those reporting one symptom (69.812; p = 0.014), two symptoms (139.225; p < 0.001), and ≥ 3 symptoms (217.503; p < 0.001) had progressively higher scores than those with none, with all pairwise comparisons statistically significant. Additionally, higher knowledge was observed among participants whose fathers (p < 0.001) or mothers (p < 0.001) were literate, those consuming a non-vegetarian diet (p < 0.001), and those with normal nutritional status (p = 0.001).

Knowledge regarding WIFS and deworming was significantly associated with age, educational grade, caste, number of anaemia-related symptoms, gender, per capita monthly income (PCMI), parental literacy, and dietary practices (p < 0.001). Participants aged 15 years had significantly higher knowledge than those aged < 14 years (93.890; p = 0.001) and 14 years (43.486; p = 0.033), while those aged ≥16 years scored higher than participants aged <14 years (131.340; p < 0.001) and 14 years (80.936; p = 0.001). Students in Standards 9 and 10 had greater knowledge than those in Standard 8 (82.046 and 95.584; both p < 0.001). OBC (133.651; p < 0.001) and SC participants (90.282; p < 0.001) had higher scores than the General category. Knowledge increased with the number of reported anaemia symptoms: Those with one (61.704; p = 0.029), two (171.737; p < 0.001), and ≥ 3 symptoms (190.182; p < 0.001) had significantly higher scores than those with none. Participants from the lowest PCMI quartile (Q1) had lower knowledge than those in Q2 (76.191; p = 0.005), Q3 (66.263; p = 0.006), and Q4 (82.393; p < 0.001). Male participants had higher WIFS knowledge than females (p < 0.001). Higher knowledge was also associated with literate parents (father and mother: both p < 0.001) and a non-vegetarian diet (p = 0.001), but not with nutritional status.

**Figure 3:**
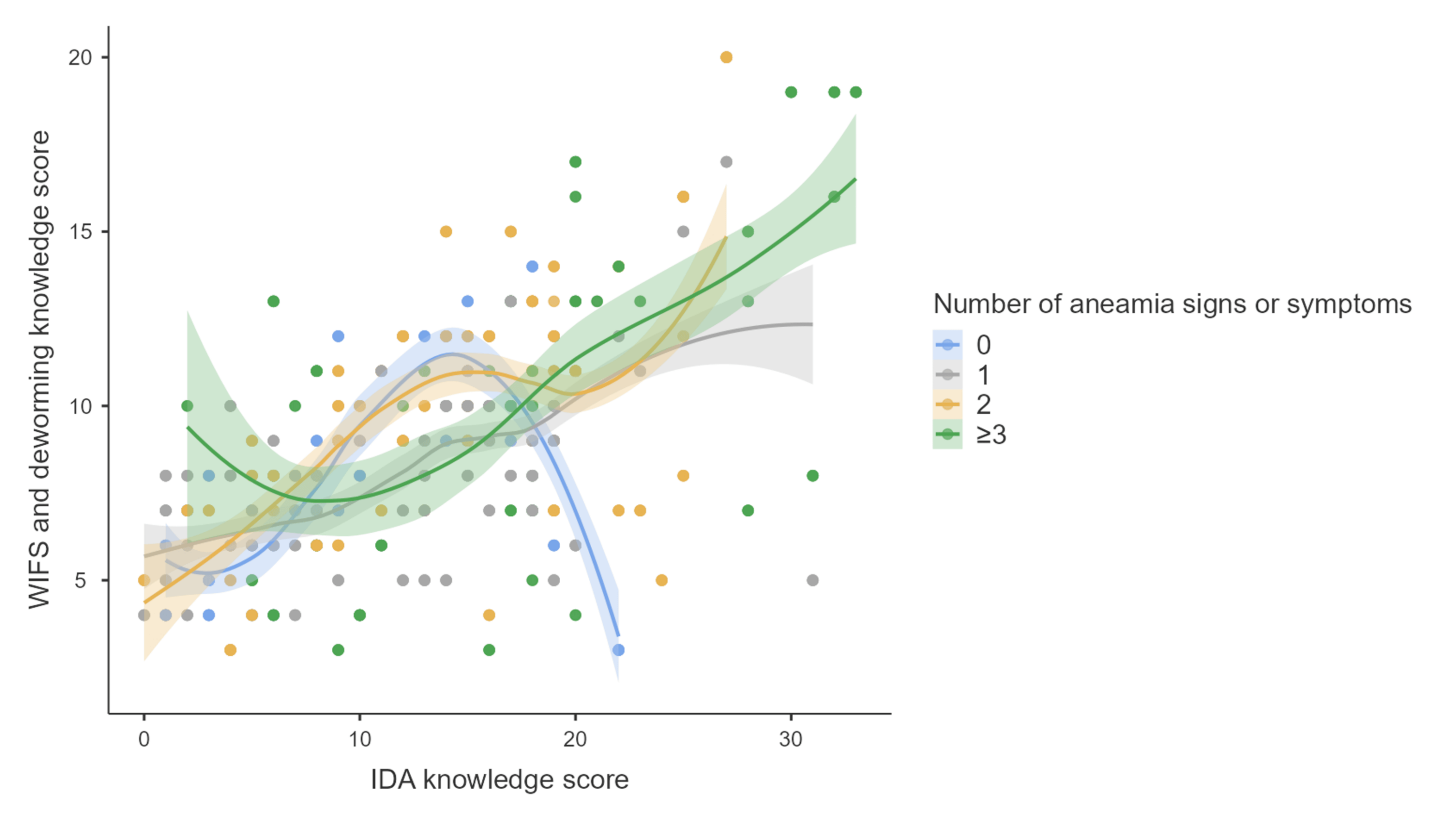
Scatter plot depicting the distribution of study participants by the number of reported anaemia-related signs or symptoms and their knowledge scores on IDA, WIFS, and deworming (n = 843) IDA: Iron deficiency anaemia; WIFS: Weekly iron and folic acid supplementation

**Figure 4:**
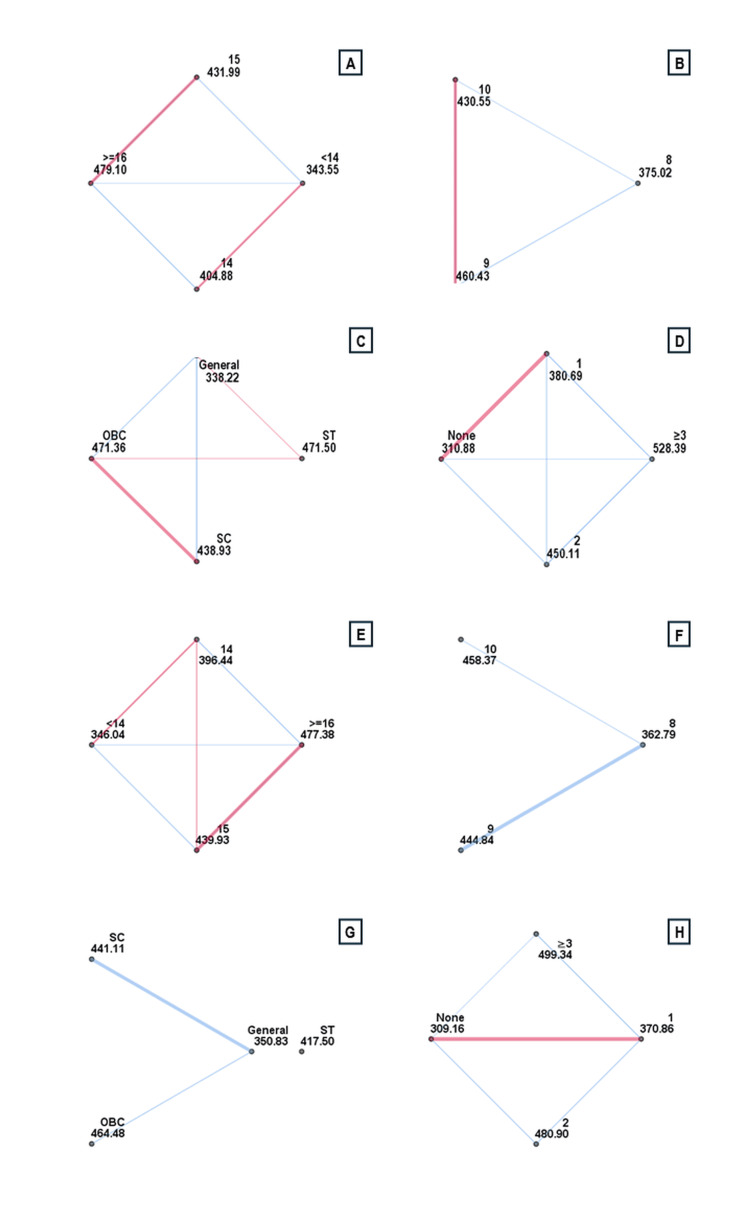
Pairwise comparison of study participants based on age, reading standard, caste, number of reported signs or symptoms of anaemia and their knowledge scores related to IDA (n = 843) Statistical analysis was performed using the Kruskal–Walli’s test. A: IDA awareness score by age; B: IDA awareness score by reading standard; C: IDA awareness score by caste; D: IDA awareness score by number of reported signs or symptoms of anaemia. E: WIFS and deworming awareness score by age; F: WIFS and deworming awareness score by reading standard; G: WIFS and deworming awareness score by caste; H: WIFS and deworming awareness score by number of reported signs or symptoms of anaemia IDA: Iron deficiency anaemia; WIFS : Weekly iron and folic acid supplementation; OBC: Other Backward Class; SC: Scheduled Caste; ST: Scheduled Tribe

There was a significant positive correlation between IDA and WIFS-deworming knowledge scores (Spearman’s ρ = 0.546, p < 0.001). IDA knowledge was also significantly correlated with the number of anaemia-related signs or symptoms (ρ = 0.269, p < 0.001), as was WIFS and deworming knowledge (ρ = 0.276, p < 0.001) (Table 2, Figures 3, 4).

**Table 2:** Distribution of the study participants as per their background characteristics and knowledge regarding IDA (n = 843) ^*^Mann–Whitney U test; ^#^Kruskal–Walli’s test IDA: Iron deficiency anaemia; IQR: Interquartile range; OBC: Other Backward Class; SC: Scheduled Caste; ST: Scheduled Tribe; PCMI: Per capita monthly family income; Q: Quartile

Variable	N (%)	Knowledge Score Regarding IDA Median (IQR)	Test Statistic	p-value	Knowledge Score Regarding WIFS and Deworming Median (IQR)	Test Statistic	p-value
School:							
1	215 (25.5)	13 (7-18)	0.642	0.887^#^	8 (6-11)	1.513	0.679^#^
2	224 (26.6)	14 (8-18)			8 (6-11)		
3	196 (23.3)	14 (7-19)			9 (7-11)		
4	208 (24.7)	14 (6-19)			9 (6-11)		
Age in completed years:							
< 14	104 (12.3)	10 (6-17)	22.197	<0.001^#^	7 (5-9)	23.866	<0.001^#^
14	272 (32.3)	13 (6-18)			8 (6-11)		
15	294 (34.9)	14 (6-19)			9 (6-11)		
≥ 16	173 (20.5)	15 (9-19)			9 (7-11)		
Gender:							
Male	401 (47.6)	14 (8-18)	0.065	0.948^*^	9 (7-11)	5.674	<0.001^*^
Female	442 (52.4)	14 (6-19)			7 (6-11)		
Reading standard:							
8	281 (33.3)	12 (6-17)	17.849	<0.001^#^	8 (6-10)	25.578	<0.001^#^
9	281 (33.3)	15 (7-19)			9 (6-11)		
10	281 (33.3)	14 (8-18)			9 (7-11)		
Caste:							
General	276 (32.7)	9 (4-16)	50.652	<0.001^#^	7 (6-10)	36.912	<0.001^#^
OBC	397 (47.1)	15 (9-19)			9 (7-11)		
SC	150 (17.8)	15 (6-19)			9 (6-12)		
ST	20 (2.4)	15 (12-19)			9 (7-10)		
Religion:							
Hindu	761 (90.3)	14 (7-19)	1.178	0.239^*^	9 (6-11)	0.637	0.524^*^
Muslim	82 (9.7)	14 (7-16)			9 (6-10)		
Father's educational level:							
Illiterate	269 (31.9)	11 (6-17)	3.993	<0.001^*^	8 (6-10)	4.686	<0.001^*^
Literate	574 (68.1)	14 (8-19)			9 (7-11)		
Mother's educational level:							
Illiterate	306 (36.3)	11 (6-16)	5.669	<0.001^*^	7 (5-9)	7.269	<0.001^*^
Literate	537 (63.7)	15 (8-19)			10 (7-11)		
PCMI (in USD):							
Q1 (6.1-10.2)	179 (21.2)	15 (7-17)	6.000	0.112^#^	8 (6-10)	14.150	0.003^#^
Q2 (10.3-15.2)	147 (17.4)	15 (10-19)			9 (7-11)		
Q3 (15.3-21.4)	238 (28.2)	14 (1-18)			9 (7-11)		
Q4 (21.5-203.4)	279 (33.1)	11 (6-19)			9 (6-12)		
Type of diet:							
Vegetarian	85 (10.1)	7 (3-16)	4.525	<0.001^*^	7 (4-11)	3.242	0.001^*^
Non-vegetarian	758 (89.9)	14 (8-19)			9 (7-11)		
Nutritional status:							
Normal	727 (86.2)	14 (7-19)	3.301	0.001^*^	9 (6-11)	0.143	0.887^*^
Malnourished	116 (13.8)	12 (8-16)			8 (7-12)		
Number of anaemia signs or symptoms:							
None	94 (11.2)	9 (3-17)	62.866	<0.001^#^	7 (5-9)	66.511	<0.001^#^
1	331 (39.3)	13 (6-17)			8 (6-10)		
2	260 (30.8)	15 (9-19)			10 (7-12)		
≥ 3	158 (18.7)	17 (10-22)			10 (7-13)		

## Discussion

This cross-sectional study assessed knowledge related to IDA, WIFS, and deworming among school-going adolescents in four coeducational government schools in Deoghar, Jharkhand, using multistage sampling. While every second student identified iron-rich foods as preventive, only one in every nine surveyed knew that walking barefoot could lead to worm infestation and anaemia. About one in four participants recognized that anaemia could cause menstrual problems or reduce immunity. Knowledge levels were higher among older students, those in higher grades, and those with literate parents, normal nutritional status, or non-vegetarian diets. While nearly nine in ten students recognized the benefits of deworming tablets, only about one in three correctly identified the recommended intake schedule, and just one in six were aware of common side effects such as stomach pain.

In the present study, knowledge regarding the causes of anaemia ranged from 10.3% to 53.1%, which was considerably lower than that reported by Sasmita et al. (84.3%) [20]. Awareness of iron-rich food sources varied between 26.8% and 61.0%, which was comparable to findings from Salam et al. in northern Karnataka (19.4-46.0%) and Verma et al. in western Rajasthan (4.2-56.5%) but notably higher than Subba et al. in Anantapur, Andhra Pradesh (11.6%) [12,13,19]. However, these figures were still lower than those reported by Sasmita et al. (83.5%) [20].

Knowledge regarding dietary practices influencing iron absorption, such as avoiding tea or coffee after meals (63.5%) and consuming vitamin C-rich foods (54.7%), was substantially higher in our study than that reported by Subba et al. (29.2% and 19.5%, respectively) [12]. Recognition of anaemia’s impact on academic performance was also greater in our study (45.8%) compared to Salam et al. (13.6%) [19]. Conversely, awareness of anaemia’s immunological consequences was lower (32.7%) than that observed by Salam et al. (45.0%), possibly due to limited health messaging on systemic effects [19].

Regarding WIFS and deworming, 36.3% of participants knew that iron tablets should be taken weekly similar to Salam et al. (34.6%) [19]. Awareness that IFA tablets may cause black stools was 24.9% in our study, higher than Salam et al. 18.6%) [19]. Knowledge that IFA should be taken after meals (37.7%) was again comparable to Salam et al. (34.1%) [19]. More than half (56.2%) were aware of the biannual deworming schedule, exceeding the proportion reported by Salam et al. (36.1%) [19].

In the present study, knowledge regarding IDA, WIFS, and deworming was significantly higher among older adolescents, which aligns with the findings of Subba et al. and Chainisha et al. from Guntur district of Andra Pradesh, where age was also associated with better awareness [12,14]. We also observed that students in higher standards had greater knowledge, similar to the results reported by Chainisha et al. [14]. Participants with literate parents, especially mothers, had significantly higher knowledge scores in our study, consistent with Subba et al. and Chainisha et al., both of whom identified maternal education as a key determinant [12,14]. A significant association between PCMI and knowledge was also observed in our study, which parallels findings by Chainisha et al. [14]. Additionally, adolescents consuming a non-vegetarian diet had better knowledge scores, in line with Chainisha et al., who reported a significant association between dietary pattern and anaemia awareness [14].

Variations in findings across studies may be attributed to differences in participant profiles and study designs. Our study included both male and female students from Standards 8 to 10, whereas Sasmita et al. focused on students from Standards 5 and 6, Salam et al. on Standards 6 and 7, and Verma et al., Chainisha et al., and Subba et al. exclusively on adolescent girls [12-14,20]. Moreover, Subba et al. and Verma et al. employed a cross-sectional design, while Salam et al. and Sasmita et al. conducted pre-post interventional studies [12,13,19,20]. The inclusion of older students in our study likely contributed to higher knowledge scores, possibly due to increased cognitive maturity and greater exposure to curriculum-based health content. The use of a structured, validated questionnaire and data collection within schools that had ongoing WIFS and deworming programmes likely facilitated better recall and understanding of related content. The strong correlation observed between IDA and WIFS and deworming knowledge scores (ρ = 0.546, p < 0.001) suggested that these topics were often delivered together through integrated school-based messaging. Additionally, knowledge scores increased with the number of self-reported anaemia symptoms (IDA: ρ = 0.269; WIFS: ρ = 0.276; both p < 0.001), indicating that students experiencing symptoms may have paid closer attention to relevant health information.

This study has certain limitations that should be acknowledged. First, it was conducted in four government-run, coeducational schools in a single district, which may limit the generalizability of findings to other settings such as private institutions, rural tribal areas, or out-of-school adolescents. Second, although the questionnaire demonstrated high internal consistency, the reliance on self-reported responses may have led to recall bias or overestimation of knowledge, particularly in domains where personal experience with symptoms may influence responses. Third, due to the cross-sectional design, the observed associations, such as higher knowledge among students with more anaemia-related symptoms or those with better nutritional status, should be interpreted as correlational rather than causal.

## Conclusions

This study found that school-going adolescents in Deoghar had limited knowledge of IDA, WIFS, and deworming. While awareness of dietary prevention was relatively common, understanding of parasitic causes, correct tablet schedules, and side effects was low. Knowledge was significantly higher among older students, those in higher grades, with literate parents, more anaemia-related sign or symptoms, non-vegetarian diets, and normal nutritional status based on BMI. These findings highlight the need for targeted school-based education to address key gaps in anaemia prevention and supplementation awareness.

## Appendices

Appendix 1 

**Table 3:** IDA, WIFS, and deworming knowledge questionnaire IDA: Iron deficiency anaemia; WIFS: Weekly iron and folic acid supplementation; IFA: Iron folic acid

Item	Variable	Response	Score
	Knowledge regarding IDA		
	What do you know about the symptoms of anaemia?		
K1	Feeling tired is a symptom of anaemia	True	1
		False	0
		Don’t Know	0
K2	Anaemia can cause difficulty in concentrating	True	1
		False	0
		Don’t Know	0
K3	Loss of appetite can be a sign of anaemia	True	1
		False	0
		Don’t Know	0
K4	Passing worms in the stool can be related to anaemia	True	1
		False	0
		Don’t Know	0
K5	People with anaemia may feel their heart beating fast (palpitations)	True	1
		False	0
		Don’t Know	0
	How can you recognize someone who has anaemia?		
K6	People with anaemia often feel weak	True	1
		False	0
		Don’t Know	0
K7	Anaemia can cause paleness in the skin, tongue, or palms	True	1
		False	0
		Don’t Know	0
K8	Spoon-shaped nails (koilonychia) can be a sign of anaemia	True	1
		False	0
		Don’t Know	0
K9	Anaemic people may fall sick more often	True	1
		False	0
		Don’t Know	0
	What do you know about the effects of anaemia on adolescents?		
K10	Anaemia can affect your physical growth	True	1
		False	0
		Don’t Know	0
K11	It can lead to poor performance in school	True	1
		False	0
		Don’t Know	0
K12	Anaemia can reduce your ability to fight infections	True	1
		False	0
		Don’t Know	0
K13	It may cause menstrual problems in girls	True	1
		False	0
		Don’t Know	0
	What are the causes of iron deficiency anaemia?		
K14	Not eating enough iron-rich foods can cause anaemia	True	1
		False	0
		Don’t Know	0
K15	Infections like malaria or worm infestation can lead to anaemia	True	1
		False	0
		Don’t Know	0
K16	Heavy bleeding during periods may cause anaemia	True	1
		False	0
		Don’t Know	0
K17	Walking barefoot can increase the chance of getting worm infestation	True	1
		False	0
		Don’t Know	0
K18	Not taking iron tablets (IFA) can lead to anaemia	True	1
		False	0
		Don’t Know	0
K19	Not taking deworming medicine (albendazole) can increase anaemia risk	True	1
		False	0
		Don’t Know	0
	How can anaemia be prevented?		
K20	Eating iron-rich foods helps prevent anaemia	True	1
		False	0
		Don’t Know	0
K21	Eating fruits rich in vitamin C helps the body absorb iron	True	1
		False	0
		Don’t Know	0
K22	Taking iron tablets regularly can prevent anaemia	True	1
		False	0
		Don’t Know	0
K23	Treating infections early helps in preventing anaemia	True	1
		False	0
		Don’t Know	0
	What are some good sources of iron?		
K24	Green leafy vegetables (like spinach) are rich in iron	True	1
		False	0
		Don’t Know	0
K25	Jaggery (gur) is a good source of iron	True	1
		False	0
		Don’t Know	0
K26	Whole grains (like wheat, bajra) contain iron	True	1
		False	0
		Don’t Know	0
K27	Fruits (like pomegranate, apple) are sources of iron	True	1
		False	0
		Don’t Know	0
K28	Liver (animal source) contains iron	True	1
		False	0
		Don’t Know	0
K29	Fish is a good source of iron	True	1
		False	0
		Don’t Know	0
K30	Pulses and legumes (like lentils, rajma) are rich in iron	True	1
		False	0
		Don’t Know	0
K31	Nuts (like almonds, groundnuts) contain iron	True	1
		False	0
		Don’t Know	0
	What helps or harms iron absorption and adolescent needs?		
K32	Drinking tea or coffee after meals reduces iron absorption	True	1
		False	0
		Don’t Know	0
K33	Eating vitamin C-rich foods (like lemon or amla) helps absorb iron	True	1
		False	0
		Don’t Know	0
K34	Teenagers need more iron and folic acid than children or adults	True	1
		False	0
		Don’t Know	0
K34	Teenagers need more iron and folic acid than children or adults	True	1
		False	0
		Don’t Know	0
	Knowledge regarding WIFS and Deworming		
	How should iron tablets (IFA) AND deworming tablets (albendazole) be taken in school?		
K35	How often should the iron tablet (IFA) given in school be taken?	Monthly	0
		Yearly	0
		Weekly	1
		Don’t Know	0
K36	When should the iron tablet (IFA) be taken?	Before Food	0
		After Food	1
		Anytime	0
		Don’t Know	0
K37	How often should the deworming tablet (albendazole) be taken in school?	Monthly	0
		Twice a year (Bi-annually)	1
		Annually	0
		Don’t Know	0
	What are the benefits of taking iron tablets (IFA)?		
K38	Iron tablets (IFA) have no benefit	True	0
		False	1
		Don’t Know	0
K39	Iron tablets help improve concentration	True	1
		False	0
		Don’t Know	0
K40	Iron tablets help you feel healthier	True	1
		False	0
		Don’t Know	0
K41	Iron tablets help in gaining weight	True	1
		False	0
		Don’t Know	0
K42	Iron tablets reduce tiredness	True	1
		False	0
		Don’t Know	0
	What are the benefits of taking deworming tablets (albendazole)?		
K43	Deworming tablets have no benefit	True	0
		False	1
		Don’t Know	0
K44	Deworming tablets kill stomach worms	True	1
		False	0
		Don’t Know	0
K45	Deworming tablets kill filaria parasites	True	1
		False	0
		Don’t Know	0
	What are the common side effects of iron tablets (IFA)?		
K46	Iron tablets have no side effects	True	0
		False	1
		Don’t Know	0
K47	Iron tablets can make stool look black	True	1
		False	0
		Don’t Know	0
K48	Iron tablets can cause a metallic taste in mouth	True	1
		False	0
		Don’t Know	0
K49	Iron tablets can make the stomach feel full	True	1
		False	0
		Don’t Know	0
K50	Iron tablets can cause constipation	True	1
		False	0
		Don’t Know	0
K51	Iron tablets can cause stomach pain	True	1
		False	0
		Don’t Know	0
K52	Iron tablets can cause loose motion (diarrhoea)	True	1
		False	0
		Don’t Know	0
K53	Iron tablets can cause nausea	True	1
		False	0
		Don’t Know	0
K54	Iron tablets can cause headache	True	1
		False	0
		Don’t Know	0
